# Histomorphometric study of brachiocephalic artery of Japanese quail

**Published:** 2015-09-15

**Authors:** Sarah Shariati, Farhad Rahmanifar, Amin Tamadon

**Affiliations:** 1 *Department of Basic Sciences, School of Veterinary Medicine, Shiraz University, Shiraz, Iran; *; 2 *Transgenic Technology Research Center, Shiraz University of Medical Sciences, Shiraz, Iran.*

**Keywords:** Age, Brachiocephalic artery, Japanese quail, Sex, Histomorphometry

## Abstract

Brachiocephalic arteries in quails are large arteries which are arising separately from the aortic arch. The aim of the present study was to determine the histomorphometric aspects of brachiocephalic arteries in the Japanese quail. The different layers of the brachiocephalic artery were studied quantitatively in 10, 20 and 60 days-old Japanese quail; (n = 6) and both sexes. Luminal diameter, thickness of the intima, media and adventitia, the percentage of the intima, media and adventitia, as compared with the total wall thickness were determined. It was found that luminal diameter and whole artery thickness increased by age (*p* < 0.05). In addition, the tunica media was the thickest layer, then tunica intima and at last tunica adventitia (*p* < 0.05). The muscularity of the right brachiocephalic artery was more than that of the left one (*p* < 0.05). Histomorphometric study of brachiocephalic arteries of Japanese quails showed that increasing of age causes increase of internal and external diameters of the artery and this increase in females was more than males.

## Introduction

The aorta and its branches are ascertained by their high elasticity. This supports them smooth out the extensive variability in blood pressure produced by the heartbeat specially when transferring blood to the wings during flight. In contrast to mammalian, in birds the second branches of aorta are brachiocephalic arteries which arise basically to the common carotid and subclavian arteries.^1^ There are differences between the avian species in the branching of the brachiocephalic trunk;^2^^,^^3^ however, it is an important vessels which distributes blood to the wing arteries.^4^ Prominent left and right brachiocephalic arteries origins from the left side of the ascending aorta are only slightly apart. The left brachiocephalic artery takes a direct course; the right brachiocephalic artery curve ventral to the ascending aorta proximal to the point at which it leaves the sac.^1^ Each brachiocephalic artery is about half the caliber of the arch of the aorta. Each brachiocephalic artery divides into subclavian and common carotid arteries after a short craniolateral course.^1^

As implied by their names, the brachiocephalic arteries are distributed to the arm and head regions. After a short craniolateral course each brachiocephalic artery divides into subclavian and common carotid arteries.^3^

Arteries are classified into elastic, transitional and muscular types based on the histological characteristics and organization of connective tissue fibers and smooth muscle cells. Like other arteries, the wall of the brachiocephalic artery is composed of three tunics. The tunica intima consists of a single layer of flatted endothelial cells on the internal elastic membrane. The tunica media completely divides of elastic lamellae and composed of predominantly smooth muscle cells with a few fine elastic tissue fibers. Tunica adventitia is mainly composed of collagen fibers.^5^^,^^6^ Martins *et al*. studied the quantitative variations of elastic and collagenous fibers, muscle cells and cellular population in the middle portion of the thoracic aorta of *Gallus gallus *from 1 day to 36 months of age.^7^ Ocal *et al*. found that the ascending aorta, the aortic arch and the thoracic aorta are elastic type in chicken.^8^ Rahmanifar *et al*. stated that the left and right brachiocephalic arteries in chicken contain elastic lamellae.^9^ They were also showed the same results in pigeon.^10^ There is little knowledge about development of various layers of brachiocephalic arteries. The purpose of this investigation was to report hitherto unknown histomorphometric data on brachiocephalic arteries of Japanese quails at different ages (neonate to adult) and both sexes (male and female). 

## Materials and Methods

**Fig. 1:**
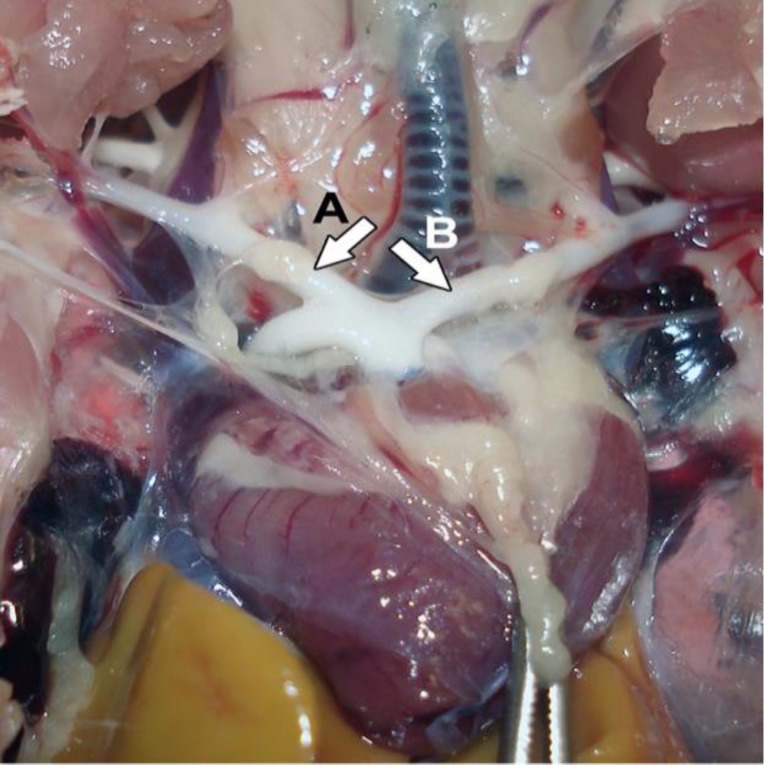
Left (A) and right (B) brachiocephalic arteries (arrows) in Japanese quail

Eighteen Japanese quails were divided into three groups of age; 10, 20 and 60 days old (n = 6). Every group contained three females and three males. They were maintained in the Animal Research Unit of the Veterinary School of Shiraz University, Shiraz, Iran. They received food and water *ad* *libitum* during the experiment. The quails were humanly euthanized by cervical dislocation. Immediately following dissection, the right and left brachiocephalic arteries (Fig. 1) were collected and fixed in a 10% formalin buffer solution. After fixation, segments were embedded in paraffin, and histological sections were made from each block. The 5 µm thickness sections were stained with hematoxylin-eosin (H&E) and green Masson’s trichrome.

Luminal diameter (internal diameter), whole artery diameter (external diameter) and wall thickness were measured. Moreover, the thickness of the tunica intima, tunica media and tunica adventitia were taken with the aid of ocular micrometer. 

Means and standard error (SE) of histomorphometric parameters of arteries were statistically compared between different groups of ages and sexes using two-way ANOVA using SPSS (Version 11.5; SPSS Inc., Chicago, USA). The *p* value less than 0.05 was considered to be statistically significant. 

## Results

**Fig. 2:**
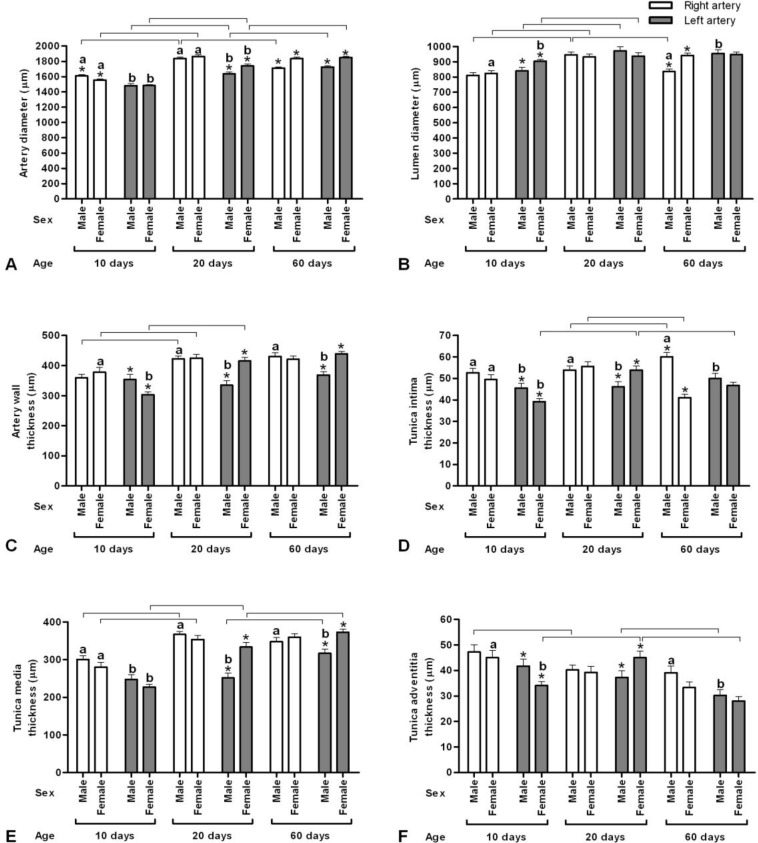
Histomorphometry (Mean ± standard error) of right and left brachiocephalic artery at different ages in male and female Japanese quail. **A)** Artery diameter (μm); **B)** Lumen diameter thickness (μm); **C)** Artery wall thickness (μm); **D)** Tunica intima thickness (μm);  **E)** Tunica media thickness (μm); **F)** Tunica adventitia thickness (μm).

Diameter of the right arteries in both sexes increased with age until day 20 (*p* < 0.05) and thereafter until day 60, it did not significantly change in female (*p* > 0.05) but in male the artery diameter decreased until day 60 (*p* = 0.0, Fig. 2A). However, the diameter of right arteries in male was more than female at day 10 (*p* = 0.03); on day 20 there was no difference between both sexes (*p* = 0.31) and on day 60 the diameter of right arteries in females was more than males, conversely (*p* = 0.0). Moreover, diameter of the left arteries in both sexes increased with age until day 60 (*p* < 0.01). However, the diameter of left arteries had not significant differences between male and female quails on day 10; with increasing age the diameter of the left arteries in females increased more than males (*p* < 0.01). In addition, in both sexes with age increasing until day 20, the diameter of the right arteries was more than the left ones (*p* < 0.01); meanwhile, on day 60 there was no different between left and right arteries (*p* > 0.05).

Lumen diameter of the right arteries in both sexes increased with age until day 20 (*p* < 0.01) and thereafter in female it had not a significant change until day 60 (*p* = 0.70) but in male the lumen diameter decreased until day 60 (*p* = 0.00, Fig. 2B). However, there was no difference between the lumen diameter of right arteries on days 10 and 20 in both sexes (*p* > 0.05), on day 60 in female was more than male (*p* = 0.00). Moreover, lumen diameter of the left arteries in both sexes increased with age until day 20 (*p* < 0.05) and thereafter until day 60 the lumen diameter had not a significant change (*p* > 0.05). 

Caps indicate statistically significant difference between columns.

^a,b^ different letters indicate statistically significant differences between same saxes in the same days.

* Stars indicate statistically significant difference between male and female in the same days (*p* < 0.05).

However, the lumen diameter of left arteries in female was more than male quails on day 10 (*p* = 0.01), the lumen diameter of left arteries had not significant differences between male and female quails on days 20 and 60 (*p* > 0.05), in female on day 10 and in male on day 60, the diameter of the left arteries was more than the right ones (*p* < 0.01). 

The wall thickness of the right arteries in both sexes increased with age until day 20 (*p* < 0.05) and thereafter until day 60 it had not a significant change in both sexes (*p* > 0.05, Fig. 2C). There was no difference between the wall thicknesses of the right arteries between both sexes in three ages (*p* > 0.05). Moreover, the wall thickness of the left arteries in female quails increased with age until day 20 (*p* = 0.0), the wall thickness of the left arteries in male quails did not change with age (*p* > 0.05). However, the wall thickness of left arteries in male was more than female quails on day 10; with increasing age the wall thickness of the left arteries in females increased more than males, conversely (*p* < 0.01). In addition, the diameter of the right arteries was more than the left ones in female quails on day 10, and in male quails on days 20 and 60 (*p* < 0.01). 

**Fig. 3:**
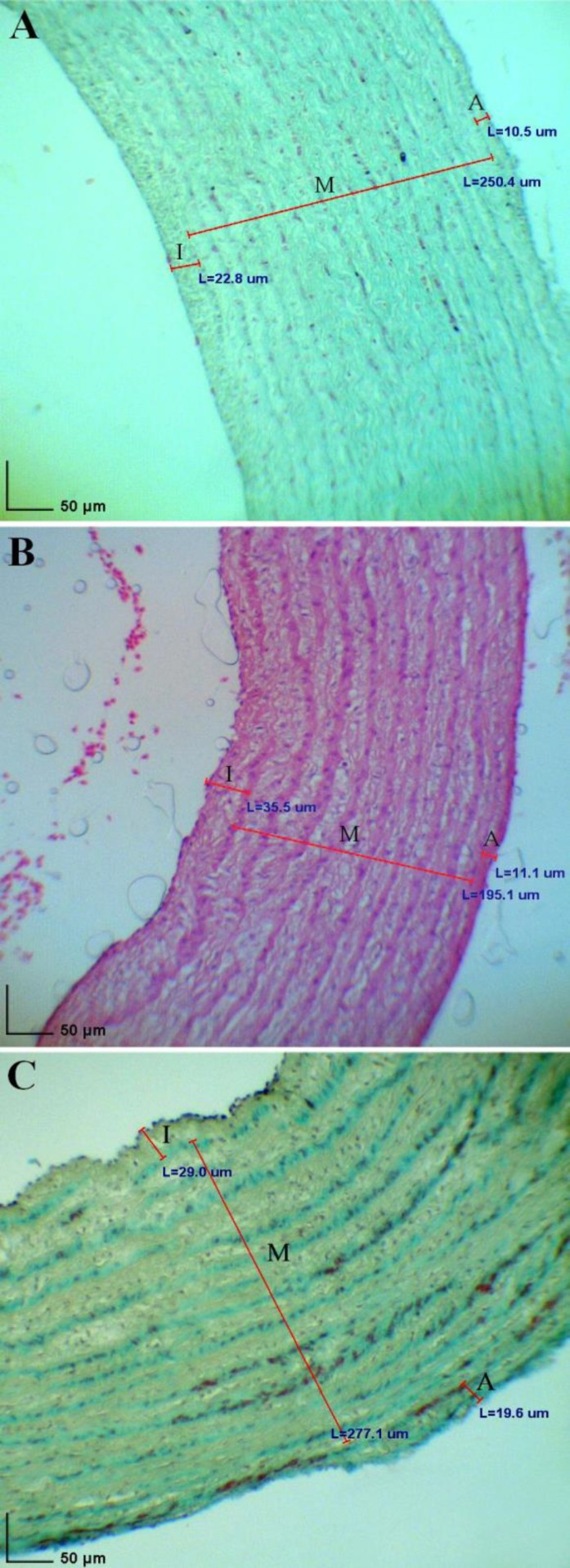
Diameter of tunics of the left brachiocephalic artery at day 10 (A, H & E), 20 (B, H & E), and 60 (C, green Masson’s trichrome staining) in female quail. **I)** Tunica intima; **M)** Tunica media; and **A)** Tunica adventitia

Tunica intima thickness of the right arteries in both sexes did not change between days 10 and 20 (*p* > 0.05) but between days 20 and 60 in male quails increased and in female ones decreased with age (*p* < 0.05, Fig. 2D). However, on day 10 and 20 there was no different in tunica intima thickness of right arteries between both sexes (*p* > 0.05), the thickness in male was more than female on day 60 (*p* = 0.0). Moreover, tunica intima thickness of the left arteries in females increased with age until day 20 and then decreased until day 60 (*p *< 0.01) but in males there was no different in tunica intima thickness of left arteries with age (*p* > 0.05). The tunica intima thickness of the left arteries in females was more than males on day 10 and less than males on day 20 (*p* < 0.01, Fig. 3) but it had not significant difference on day 60 (*p* > 0.05). In addition, in males on day 10, 20 and 60 and in females on day 10, the tunica intima thickness of the right arteries was more than the left ones (*p* < 0.05); meanwhile, in female quails the tunica intima thickness of the left arteries was more than the right ones on day 60 (*p* = 0.01).

Tunica media thickness of the right arteries in both sexes increased with age until day 20 (*p* < 0.01) and thereafter until day 60 it had not a significant change in both sexes (*p* > 0.05, Fig. 2E). However, the tunica media thickness of right arteries was not significantly different between both sexes in different ages (*p* > 0.05). Moreover, tunica media thickness of the left arteries in females increased with age until day 60 (*p* < 0.01) and in males increased between day 20 and 60 (*p* = 0.00). However, the tunica media thickness of left arteries had not significant differences between male and female quails on day 10; with increasing age the tunica media thickness of the left arteries in females increased more than males (*p* < 0.01). In addition, in males on days 10, 20 and 60 and in females on day 10, the tunica media thickness of the right arteries was more than the left ones (*p* < 0.05). 

**Fig. 4:**
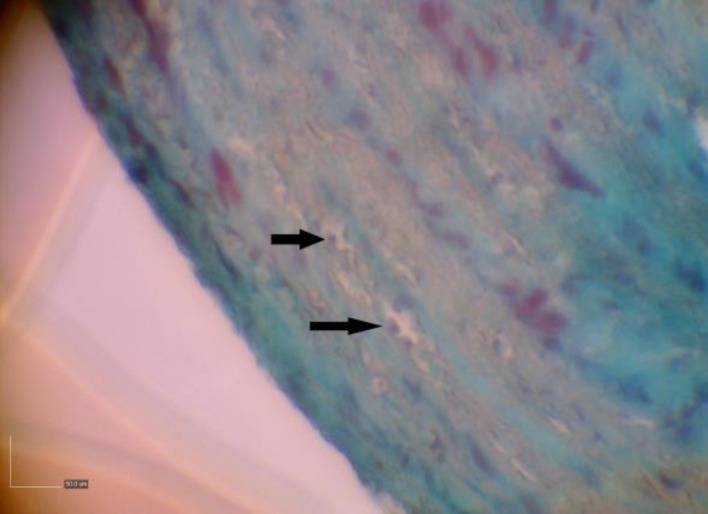
Left brachiocephalic artery at day 60 in female quail. Vasa vasorum (arrows) in tunica adventitia (Green Masson’s trichrome, 2900×).

Tunica adventitia thickness of the right arteries in females did not change with age (*p* > 0.05), but in male quails between days 10 and 20 decreased (*p* = 0.04) and did not change until day 60 (*p* = 0.72, Fig. 2F). On days 10, 20 and 60, there was no significant difference in tunica adventitia thickness of right arteries between both sexes (*p* > 0.05). Moreover, tunica adventitia thickness of the left arteries in females increased with age until day 20 and then decreased until day 60 (*p* < 0.01) but in males there was no different in tunica adventitia thickness of left arteries between days 10 and 20 (*p* = 0.26) and then decreased until day 60 (*p* = 0.04). The tunica adventitia thickness of the left arteries in males was more than females on day 10 and less than females on day 20 (*p* < 0.05) but it had not significant differences at day 60 (*p* > 0.05). In addition, in males on day 60 and in females on day 10, the tunica adventitia thickness of the right arteries was more than the left ones (*p* < 0.05). Table 1 shows the percentage of the intima, media and the adventitia, as compared with the total wall thickness. The tunica adventitia was the thinnest of the three tunics and grown partly, composed of connective tissue fibers, vasa vasorum and small nerves (Fig. 4). 

**Table 1:** Mean and standard error of percentage of the tunica intima, tunica media and tunica adventitia of right and left brachiocephalic artery as compared with the total wall thickness in Japanese quail in different ages and sexes.

**Artery layers**	**Sex**	**Day 10**		**Day 20**		**Day 60**	
		**Right**	**Left**	**Right**	**Left**	**Right**	**Left**
**Tunica intima **	Male	11.50 ± 1.70^a^	12.70 ± 0.70^A^	5.50 ± 1.10^b^*	12.20 ± 1.30^A^*	11.80 ± 1.40^a^*	6.90 ± 1.80^B^*
	Female	13.10 ± 0.60^a^	12.90 ± 0.30^A^	8.40 ± 1.60^b^	11.10 ± 1.30^AB^	8.70 ± 0.70^b^	8.50 ± 0.90^B^
**Tunica media **	Male	80.10 ± 2.20^a^	75.60 ± 1.40^A^	87.40 ± 1.40^b^*	76.40 ± 1.30^A^*	80.80 ± 1.50^a^†*	85.90 ± 1.70^B^*
	Female	77.10 ± 2.50^a^	75.90 ± 0.70^A^	83.50 ± 2.00^b^	80.40 ± 1.50^B^	85.60 ± 1.20^b^†	84.90 ± 1.00^C^
**Tunica adventitia **	Male	8.20 ± 1.70^a^	11.60 ± 0.80^A^	7.00 ± 0.80^a^	11.30 ± 0.90^A^	7.20 ± 1.10^a^	7.00 ± 0.70^B^
	Female	9.60 ± 2.00^a^	11.00 ± 0.70^A^	8.00 ± 0.90^a^	8.30 ± 1.30^A^	5.50 ± 0.80^b^	6.40 ± 0.40^B^

a,b,A,B,C Different superscript letters show significant differences between percentage of different days in the same side and the same sex in each layer (*p *< 0.05).

* Stars show significant differences between percentage of different sides in the same day and the same sex in each layer (*p *< 0.05).

† Crosses show significant differences between percentage of different sexes in the same day and the same side in each layer (*p* < 0.05).

## Discussion

Like other arteries, left and right brachiocephalic arteries of fowl contain three layers.^11^ The brachiocephalic arteries of quail were observed to be elastic type (like aorta), as previously reported in guinea pig,^6^ chicken,^9^ sheep and goat,^12^ dog,^13^ and Gottingen miniature swine.^14^ The tunica intima consisted of elongated flattened endothelial cells resting on loose areolar connective tissue. Internal elastic lamina was inconspicuous, cause of large amount of elastic lamellae in tunica media. The tunica intima of elastic arteries was thicker than muscular arteries, which are in agreement with findings in the renal artery in post-natal life of sheep.^15^ In this study, the tunica intima of Japanese quail was 13.00%, but in the mentioned study, it was 10.00%.^15^ The tunica media of the elastic arteries was well developed and consisted of high proportion of concentric layers of elastic lamellae with smooth muscle fibers. These allow the vessels to dilate. The recoil sends the blood onwards, creating the pulse in the major elastic arteries like aorta and brachiocephalic artery.^16^ Moreover, this finding was in agreement to findings in chicken,^9^ pigeon,^10^ dog,^13^ and rat.^17^ The tunica adventitia was the thinnest of the three tunics and grown partly, composed of connective tissue fibers, vasa vasorum and small nerves. Internal and external diameters of brachiocephalic artery were increased by age. This result has already been reported in chicken.^9^

In puberty age (day 20), the lumen diameter in male quails was more than female, since that lumen diameter is proportionate to the amount of blood in the artery, and in male quail, blood is more than female (cause of testosterone hormone).^18^ However, in adult quails (day 60), the lumen diameter in females is more than male quails. This increase is associated with more need for blood for egg-laying in female quail. The same finding was observed in pigeon.^10^ By increasing age, body size and surface also grow, therefore, there is more need to blood and all the constituent parts of an artery must develop. Due to the need for more contraction and dilation strength, the artery diameter is more dependent to muscular layers in tunica media. So most of the structural composition of brachiocephalic was tunica media (75.00 to 85.00% of wall thickness), then tunica intima (12.00 to 13.00%), that its amount decreased by increasing age. At last, it was the tunica adventitia (7.00 to 12.00%) which the percentage of this layer decreased by age. As tunica adventitia was the least amount of artery composition, this finding is related to those of King and McLelland^2^ and Hodges^19^ stated that the adventitia of the muscular arteries is thicker than the elastic arteries. On day 10, there were 15 layers of elastic lamellae, which by increasing age, the number of these lamellae were stable, so it could be stated that by increasing age the thickness of lamellae and muscular cells population were increased. On day 10, the artery diameter in left brachiocephalic artery of male quail was 1484.30 µm and in right artery was 1610.20 µm which this increase was significant and continued till day 60. Also on day 10, tunica intima made up 13.80%, the media 78.80% and the adventitia 12.50% in the right brachiocephalic and 12.80%, 72.40% and 11.50% in the left brachiocephalic artery. It shows that the muscularity of the right brachiocephalic artery was more than that of the left one.

## References

[1] Baumel JJ Aves, Getty R, Sisson S, Grossman’s JD (1975). Heart and Blood Vessels. The Anatomy of the Domestic Animals.

[2] King AS Phallus, King AS, Mc Lelland J Form and function in birds.

[3] Nickel R, Schummer A, Seiferle E (1977). Anatomy of the domestic birds.

[4] Neuweiler G (2000). Biology of bats.

[5] Awal MA, Prodan MA, Asaduzzaman M (1999). Histological studies on the arterial walls of main arteries supplying the mammary glands of black Bengal goats (Capra hircus) in Bangladesh. Vet Arh.

[6] Awal MA, Prodhan MAA, Kurohmaru M (2001). Microscopic studies on the arterial walls of main arteries supplying the mammary glands of guinea pig (Cavia porcellus) at different reproductive stages. Vet Arh.

[7] Martins LF, Medeiros LF, Ferri S (1970). Histometric study of age related changes in the thoracic aorta of Gallus gallus (Linnaeus, 1758). Z Anat Entwick-lungsgesch.

[8] Ocal MK, Mutus R, Corekci I (1997). A Quantitative study of the aorta of the chicken (Gallus domesticus). Anat Histol Embryol.

[9] Rahmanifar F, Firouzi S, Sharafi M (2014). Histomorphometric study of the left and right brachiocephalic arteries in different ages of chicken (Gallus domesticus). Comp Clin Pathol.

[10] Rahmanifar F, Asadi-Yosefabad SL, Sharafi M (2013). Histomorphometry of brachiocephalic artery of Iranian pigeon (Columba livia domestica). VetScan.

[11] Ball RA, Sautter JH, Katter MS (1963). Morphological characteristics of the anterior mesenteric artery of fowl. Anat Rec.

[12] Parchami A, Dehkordi RAF, Derakhshan A (2009). Comparative histomorphometric study of the common carotid artery and its terminal branches in sheep and goats. Bulg J Vet Med.

[13] Prodan MAA, Islam MR, Das SK (2001). Histological studies on the arterial walls of main arteries supplying the mammary glands of dogs (Canis familiaris) in Bangladesh. Pak J Biol Sci.

[14] Tanigawa M, Adachi J, Mochizuki K (1986). Histological study on the arterial wall of Gottingen miniature swine. Jikken Dobutsu.

[15] Gholami S, Haghighat Jahromi M (2007). Histomorphologic study of the renal artery in post-natal life of sheep (Ovis aries). Iranian J Vet Res.

[16] Aughey E, Frye FL (2001). Comparative veterinary histology with clinical correlates.

[17] Awal MA, Matsumoto M, Nishinakagawa H (1995). Morphometrical changes of the arterial walls of main arteries from heart to the abdomino-inguinal mammary glands of rat from virgin through pregnancy, lactation and post-weaning. J Vet Med Sci.

[18] Balthazart J, Schumacher M, Ottinger MA (1983). Sexual differences in the Japanese quail: behavior, morphology, and intracellular metabolism of testosterone. Gen Comp Endocrinol.

[19] Hodges RD (1974). The histology of the fowl.

